# γ-Tocotrienol Inhibits Proliferation and Induces Apoptosis via the Mitochondrial Pathway in Human Cervical Cancer HeLa Cells

**DOI:** 10.3390/molecules22081299

**Published:** 2017-08-04

**Authors:** Weili Xu, Yaqing Mi, Pan He, Shenghua He, Lingling Niu

**Affiliations:** Department of Food Science and Engineering, School of Chemistry and Chemical Engineering, Harbin Institute of Technology, Harbin 150001, China; yaqingmi@163.com (Y.M.); 16s125207@stu.hit.edu.cn (P.H.); heshenghua@hit.edu.cn (S.H.); niulingling2016@126.com (L.N.)

**Keywords:** γ-tocotrienol, cervical cancer, cell proliferation, apoptosis

## Abstract

γ-Tocotrienol, a kind of isoprenoid phytochemical, has antitumor activity. However, there is limited evidence that it has an effect on cervical cancer. In this study, the capacity to inhibit proliferation and induce apoptosis in human cervical cancer HeLa cells and the mechanism underlying these effects were examined. The results indicated that a γ-tocotrienol concentration over 30 μM inhibited the growth of HeLa cells with a 50% inhibitory concentration (IC_50_) of 46.90 ± 3.50 μM at 24 h, and significantly down-regulated the expression of proliferative cell nuclear antigen (PCNA) and Ki-67. DNA flow cytometric analysis indicated that γ-tocotrienol arrested the cell cycle at G0/G1 phase and reduced the S phase in HeLa cells. γ-tocotrienol induced apoptosis of HeLa cells in a time- and dose-dependent manner. γ-tocotrienol-induced apoptosis in HeLa cells was accompanied by down-regulation of Bcl-2, up-regulation of Bax, release of cytochrome from mitochondria, activation of caspase-9 and caspase-3, and subsequent poly (ADP-ribose) polymerase (PARP) cleavage. These results suggested that γ-tocotrienol could significantly inhibit cell proliferation through G0/G1 cell cycle arrest, and induce apoptosis via the mitochondrial apoptotic pathway in human cervical cancer HeLa cells. Thus, our findings revealed that γ-tocotrienol may be considered as a potential agent for cervical cancer therapy.

## 1. Introduction

Cancer is a major public health problem worldwide and it still represents the second leading cause of death [1]. Cancer rates could further increase by 50% to 15 million new cases by 2020, according to the World Cancer Report, the most comprehensive global examination of the disease to date [2]. It has been reported that more than 30% of human cancers could be prevented by an alternative strategy of appropriate dietary modification [3,4]. Therefore, attention is being focused on prevention as an ultimate strategy for the management of cancer [5,6]. In recent years, many studies have been carried out to investigate the potential cancer chemopreventive activities of natural phytochemicals, in particular dietary polyphenols [7].

Tocotrienols and tocopherols are two subgroups of vitamin E, each composed of four different isomers: alpha, beta, gamma and delta [8]. The basic chemical structures of tocopherols and tocotrienols include an aromatic chromanol ring and an isoprenoid chain, with tocotrienols containing three unsaturated phytyl side chains [9]. Barley, rice bran and palm oil have been reported to be rich in tocotrienol. The distribution of tocopherols in the plant kingdom is primarily α-tocopherol in green leafy plants and γ-tocopherol in non-green plant parts such as fruits and seeds [10]. Previous studies showed that tocotrienols exert more significant neuroprotective, anti-oxidant, anti-cancer and cholesterol-lowering properties than tocopherol [11,12,13]. Particularly, γ-tocotrienol is one of the most abundant forms of tocotrienol in foods, and has shown significant anticancer activity [14,15,16,17,18]. γ-Tocotrienol displays potent anticancer activity at treatment doses that have little or no effect on normal cell growth and viability [19,20].

In previous studies, data demonstrated that γ-tocotrienol inhibited the growth of breast [18,21,22], glioma [23], colon [24,25], prostate [26,27], liver [28], oral cavity [29], and gastric cancer cells [30,31] in a dose-dependent manner, and altered the expression of proteins to induce apoptosis of cancer cells. It was also reported that γ-tocotrienol displayed an anticancer effect against human cervical cancer cell lines [32,33]. γ-Ttocotrienol-inhibited proliferation in human cervical cancer cells through the upregulation of IL-6 and the down-regulation of cyclin D3, p16, and CDK6 expression in the cell cycle signaling pathway has been reported [32]. However, the effect of γ-tocotrienol on the proliferation inhibition and apoptosis induction in human cervical cancer HeLa cells and its mechanisms are still not fully understood. The objectives of this study were to evaluate the effects of γ-tocotrienol on proliferation and apoptosis in HeLa cells and to decipher the underlying molecular mechanism.

## 2. Results

### 2.1. Effect of γ-Tocotrienol on the Viability of HeLa Cells

**Figure 1:**
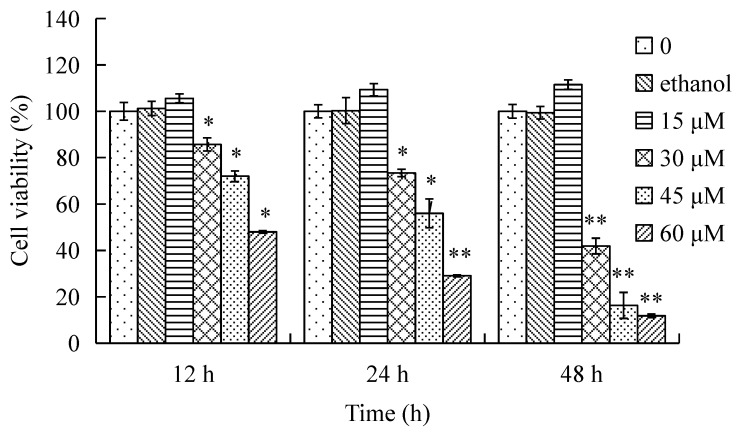
Effect of γ-tocotrienol on cell viability. HeLa cells were treated with 15, 30, 45 and 60 μM of γ-tocotrienol for 12, 24 and 48 h, and viability was determined by MTT assay. Cell viabilities are presented as percentages; the negative cells were regarded as 100% viable. Data are presented as mean ± SD (*n* = 3). * *p* < 0.05, ** *p <* 0.01, versus the control group.

**Figure 2:**
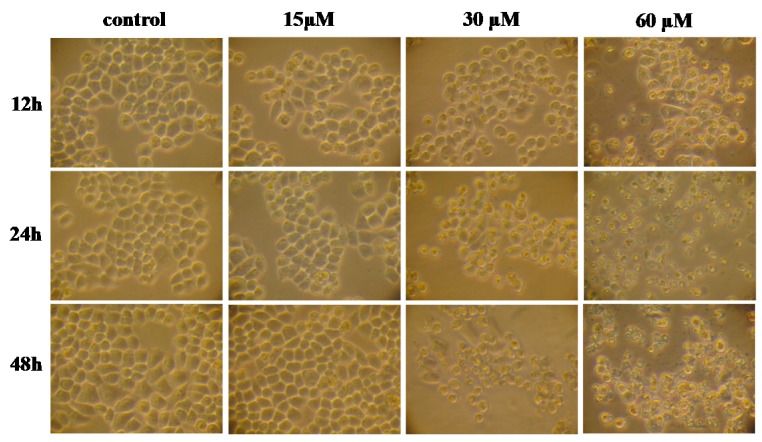
The morphological changes of HeLa cells treated by γ-tocotrienol (Inverted microscope, 100×). HeLa cells treated with 15, 30 and 60 μM of γ-tocotrienol for 12, 24 and 48 h.

The effects of γ-tocotrienol on the viability of HeLa cells are shown in Figure 1. The cells were treated with various concentrations (15, 30, 45 and 60 μM) of γ-tocotrienol for 12, 24 and 48 h. HeLa cells viability was significantly inhibited in a time- and dose-dependent manner by γ-tocotrienol above 15 μM. There was no significant difference between solvent control cells and cells treated with 15 μM of γ-tocotrienol. The 50% inhibitory concentration (IC_50_), the dose of γ-tocotrienol required to inhibit or kill 50% of the cells tested, were 59.10 ± 5.70, 46.90 ± 3.50 and 18.40 ± 1.90 μM at 12, 24 and 48 h. The differences were more clearly evident under an inverted microscope (Figure 2). HeLa cells treated with 30–60 μM of γ-tocotrienol showed shrinkage, rounding, and refractile morphology; fragmentation was even observed in some cells, in contrast to the control group.

### 2.2. Effect of γ-Tocotrienol on Mitotic Index of HeLa Cells

The effect of γ-tocotrienol treatment on mitotic index of HeLa cells is presented in Table 1. After treatment with 15 μM of γ-tocotrienol for 12 h, 24 h or 48 h, the cell mitotic index was increased compared with the control group. When the concentration of γ-tocotrienol was over 15 μM, the mitotic index was decreased in comparison with the control group in a time- and dose-dependent manner. The lowest mitotic index was observed in HeLa cells supplemented with 60 μM of γ-tocotrienol (Table 1). The inhibitions (percentages) of mitosis were 8.4–36% at 12 h, 13.1–60.2% at 24 h, and 19.5–79.2% at 48 h.

### 2.3. Effect of γ-Tocotrienol on Colony Formation in HeLa Cells

The effect of γ-tocotrienol treatment on colony formation of HeLa cells is presented in Table 2. γ-tocotrienol decreased colony formation by HeLa cells compared with controls. Inhibition ranged from 7.6% to 99.6% at 12 h, from 29.8% to 100% at 24 h and from 50.4% to 100% at 48 h after treatment with 30, 45 and 60 μM of γ-tocotrienol. These results showed that 30–60 μM of γ-tocotrienol significantly inhibited colony formation in HeLa cells in a time- and dose-dependent manner (*p <* 0.05).

### 2.4. γ-Tocotrienol Induces Cell-cycle Arrest in HeLa Cells

The cell cycle distribution of HeLa cells treated with γ-tocotrienol was determined by flow cytometry. As shown in Table 3 and Table 4, HeLa cells treated with 30, 45 and 60 μM of γ-tocotrienol for 12 and 24 h resulted in a significant increase of the proportion in G1/G0 phase and a decrease of the proportion in S phase. The proportion in G1/G0 phase increased from 61.27% to 72.03% and from 63.75% to 75.87% at 12 and 24 h, respectively. The percentage in S phase decreased from 19.84% to 8.88% and from 27.14% to 15.92% at 12 and 24 h, respectively. However, no changes in 15 μM γ-tocotrienol treatment group, solvent and the control group were observed after 12 or 24 h. These results demonstrated that 30–60 μM of γ-tocotrienol resulted in a significant increase of the proportion of cells at the G1/G0 phase, and a decrease in the proportion at S phase, in a time- and dose-dependent manner (*p <* 0.05).

### 2.5. γ-Tocotrienol Induces Apoptosis in HeLa Cells

**Figure 3:**
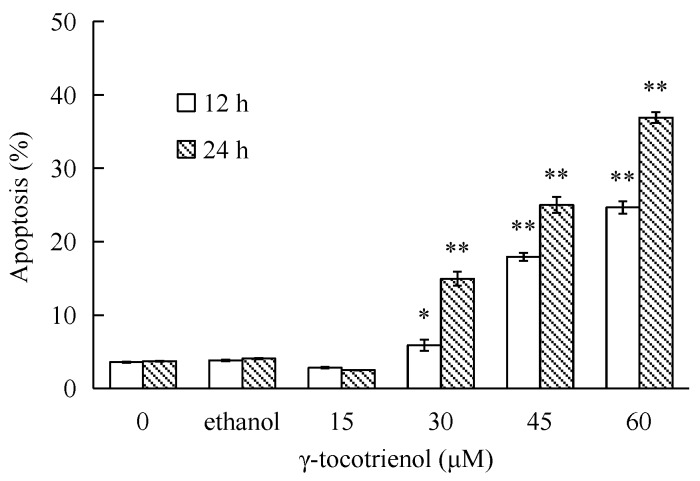
Percentage of apoptotic HeLa cells induced by different concentrations of γ-tocotrienol. HeLa cells were treated with 15, 30, 45 and 60 μM of γ-tocotrienol for 12 and 24 h, then the apoptosis rates were analyzed by flow cytometry. * *p <* 0.05, ** *p <* 0.01, compared to the negative control group.

**Figure 4:**
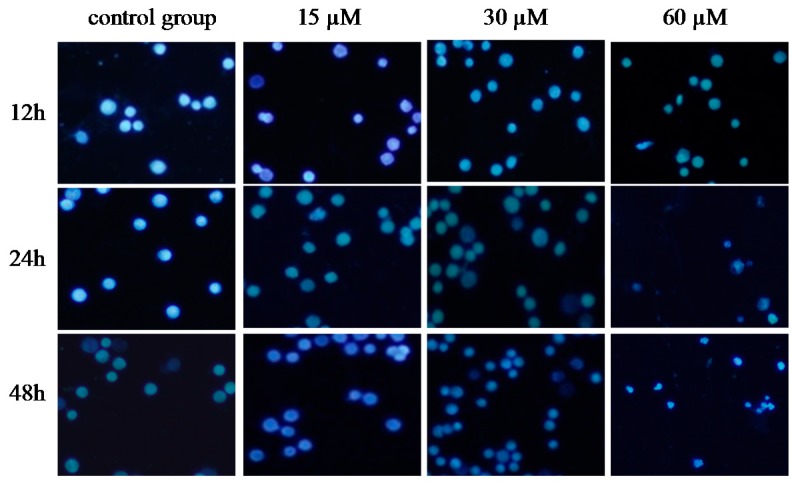
Nuclear morphological changes of HeLa cells treated by γ-tocotrienol. The cells were harvested and washed twice with phosphate-buffered saline (PBS). 25 μL of cell suspension was mixed with 1 μL of dyeing solution (4′,6-diamidino-2-phenylindole, DAPI), and the images were captured by fluorescence microscopy (Olympus IX70, 200×). Control cells showed homogeneous staining of the nucleus. After treatment with 30 and 60 μM of γ-tocotrienol for 12, 24 and 48 h, apoptotic cells had irregularly stained nuclei indicative of chromatin condensation and nuclear fragmentation.

**Figure 5:**
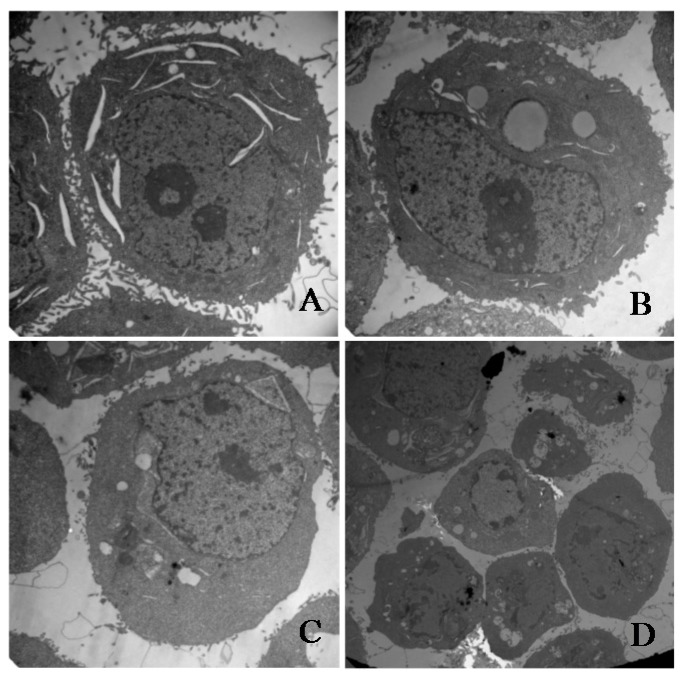
Transmission electron microscopy images. (**A**) Untreated HeLa cells (original magnification 10,000×). (**B**) HT-29 cells treated with 30 μM of γ-tocotrienol for 48 h. The treated cells became small and round. The nuclear volume decreased (original magnification 10,000×). (**C**) HT-29 cells treated with 60 μM of γ-tocotrienol for 12 h. Chromatin condensation, formation of apoptotic body and mitochondrial swelling were also observed in those cells (original magnification 10,000×). (**D**) HT-29 cells treated with 60 μM of γ-tocotrienol for 24 h (original magnification 5000×). The cells showed the chromatin condensation and disappearance of mitochondrial.

To investigate whether γ-tocotrienol-mediated growth inhibition is associated with apoptosis, treated and untreated HeLa cells were analyzed by flow cytometry. As shown in Figure 3, the apoptosis rates of HeLa cells treated with 30, 45 and 60 μM of γ-tocotrienol was 5.91–24.67% at 12 h and 15.87–36.92% at 24 h, respectively. The number of apoptotic cells in 15 μM γ-tocotrienol treatment group, solvent group was nearly the same as that of control group. In addition, DAPI staining was used to investigate the morphological changes of the cell nuclei and the results are presented in Figure 4. Control cells showed homogeneous staining of the nucleus, but, after treatment with 30 and 60 μM of γ-tocotrienol for 12, 24 and 48 h, apoptotic cells had irregularly stained nuclei indicative of chromatin condensation and nuclear fragmentation. No significant change was observed in HeLa cells treated with 15 μM of γ-tocotrienol. The ultrastructural changes are characteristic of apoptosis and further details are shown in Figure 5. The cells treated with 60 μM γ-tocotrienol for 12 and 24 h showed obvious characteristic changes of apoptosis, including cytoskeletal disruption, chromatin condensation and margination of nucleus, formation of apoptotic bodies and mitochondrial swelling, or disappearance of mitochondria (Figure 5C,D). Similarly, the characteristic morphology of apoptosis was also observed in those cells treated with 30 μM of γ-tocotrienol for 48 h (Figure 5B). In addition, cells in the control group had the clear cell organs in cytoplasm, and mitochondrial cristae were also observed clearly (Figure 5A). These data demonstrated that γ-tocotrienol significantly induced apoptosis in HeLa cells in a time- and dose-dependent manner.

### 2.6. DNA Fragmentation

**Figure 6:**
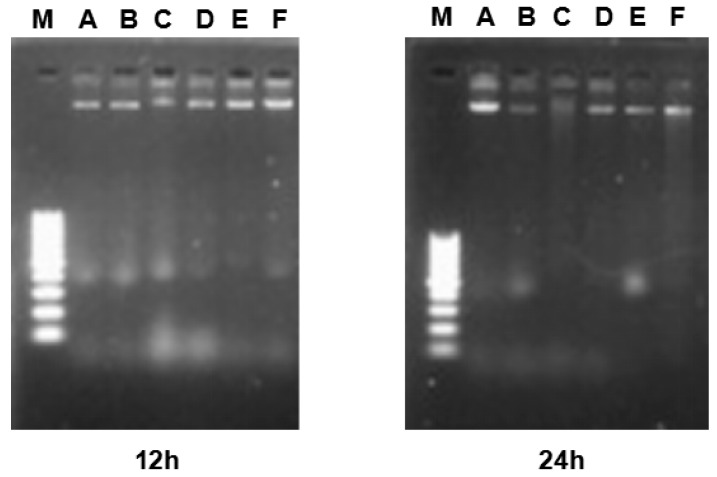
Image of agarose gel electrophoresis. (**A**) control group. (**B**) Vehicle control group. (**C** to **F**) HeLa cells were treated with 15, 30 and 60 μM for 12 and 24 h. M: Marker. DNA was isolated and subjected to 1.2% agarose gel electrophoresis, followed by visualization of bands and photography. DNA fragments were not observed in the γ-tocotrienol-treated groups and control group.

The effect of γ-tocotrienol on apoptosis in HeLa cells was further assessed by a DNA fragmentation assay. As shown in Figure 6, no DNA ladders were observed in the control group or in cells supplemented with various concentration of γ-tocotrienol for 12 and 24 h, indicating that γ-tocotrienol treated HeLa cells did not form DNA fragments. Our previous studies have shown that significant DNA ladder bands appeared in HT-29 [24] and SGC-7901 [30] cells treated with 45 and 60 μM of γ-tocotrienol for 48, 36 or 72 h.

### 2.7. Effect of γ-Tocotrienol on the Expression of Proteins Involved in Proliferation in HeLa Cells

**Figure 7:**
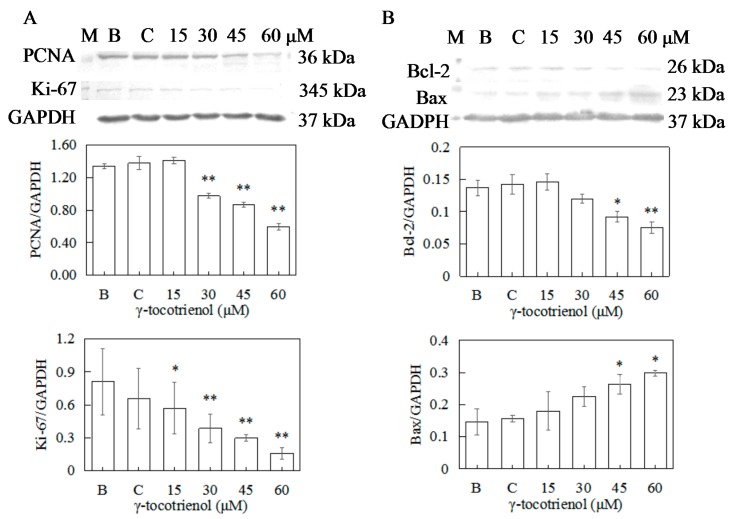
Effect of γ-tocotrienol on expression of proliferation and apoptosis-related proteins in HeLa cells. Cells were treated with various concentrations of γ-tocotrienol for 24 h. The expression levels of PCNA and Ki-67 (**A**), Bcl-2 and Bax (**B**) were analyzed by Western blot method. * *p* < 0.05, ** *p* < 0.01, compared to the negative control group (*n* = 3). M: Marker; B: Blank control; C: Ethanol control.

To confirm the antiproliferation activity of γ-tocotrienol toward human cervical cancer HeLa cells, the expressions of PCNA and Ki-67 were measured by Western blotting. As shown in Figure 7A, the expression levels of PCNA of HeLa cells treated with 15 μM of γ-tocotrienol and solvent group for 12 h did not change in comparison with the negative control. γ-tocotrienol (45 or 60 μM) significantly inhibited the expression of PCNA and Ki-67 in HeLa cells in comparison with the control group in a dose response.

### 2.8. Effect of γ-Tocotrienol on the Expression of Apoptosis-Related Proteins in HeLa Cells

**Figure 8:**
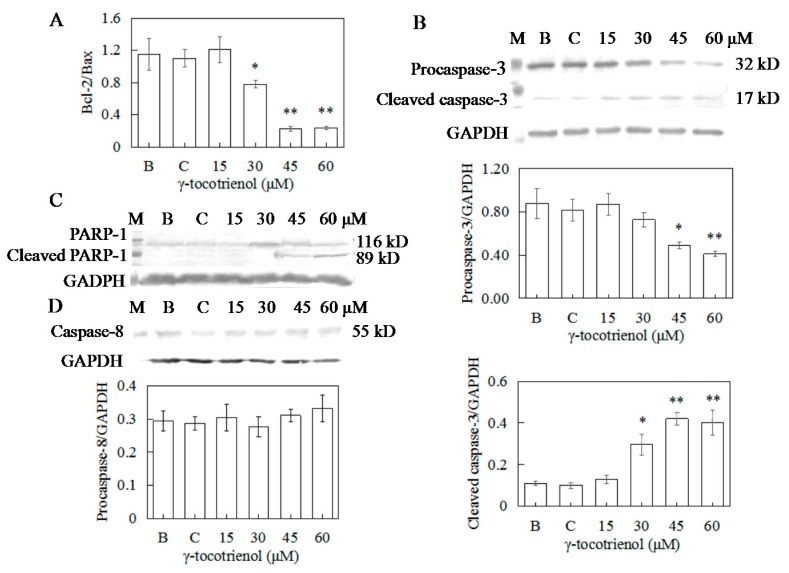
Effects of γ-tocotrienol on expression of bcl-2/bax (**A**), Caspase-3 (**B**), PARP-1 (**C**), and Caspase-8 (**D**) in HeLa cells. Cells were treated with various concentrations of γ-tocotrienol for 24 h. The expression levels of the proteins were analyzed through Western blot method. * *p* < 0.05, ** *p* < 0.01, compared to the negative control group (*n* = 3). M: Marker; B: Blank control; C: Ethanol control.

To elucidate the molecular mechanism of γ-tocotrienol induced apoptosis in HeLa cells, expression levels of apoptotic-regulation proteins such as Bcl-2, Bax, caspase-3, and PARP were evaluated. As shown in Figure 7B and Figure 8A, the expression levels of Bax and Bcl-2 were not changed in comparison with the control group when the cells were treated with γ-tocotrienol at 15 μM for 24 h. There were significant differences in the expression of Bax and Bcl-2 in HeLa cells treated with γ-tocotrienol at the dose of 45 and 60 μM in comparison with the control group (* *p <* 0.05, ** *p <* 0.01). Thus, the results indicated that γ-tocotrienol-mediated apoptosis in HeLa cells may depend on the overexpression of Bax and suppression of Bcl-2.

In response to apoptotic stimuli, procaspase-3 is cleaved into an active 17-kDa fragment. Activated caspase 3 cleaves the 116 kDa PARP protein into 89 kDa fragments. Thus, cleavage of PARP is an important marker of caspase 3-mediated apoptosis. In order to obtain direct evidence showing the relationship of caspase activation and apoptosis, procaspase-3 cleavage and PARP were examined in HeLa cells treated with γ-tocotrienol for 24 h. As shown in Figure 8B,C, the expression of cleaved caspase-3 in HeLa cells treated with 15–60 μM γ-tocotrienol for 24 h showed an increased trend. An increase of the 89-kDa cleavage form of PARP was also observed in the cells treated with 45 or 60 μM of γ-tocotrienol compared to the control group. The results indicated that γ-tocotrienol induced the cleavage of 32 kDa procaspase-3 into its active 17 kDa form and cleavage of PARP appeared in HeLa cells.

### 2.9. Apoptosis of HeLa Cells Induced by γ-Tocotrienol via Mitochondrial Pathway

**Figure 9:**
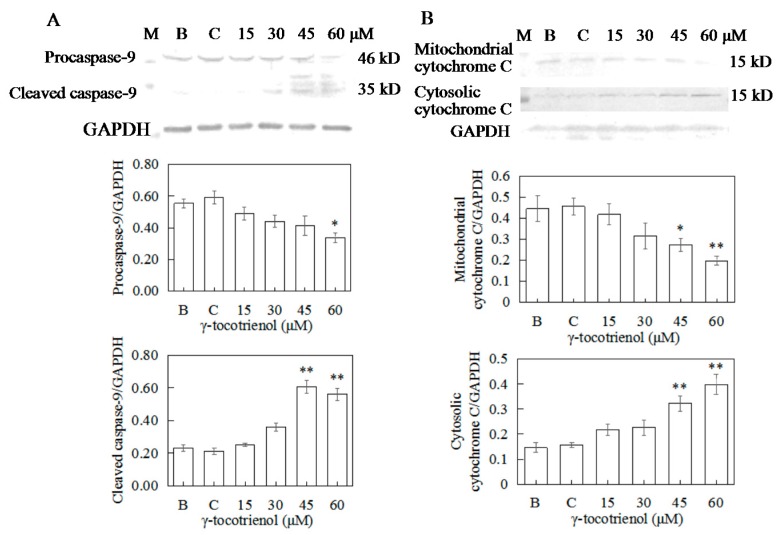
Effects of γ-tocotrienol on expression of caspase-9 (**A**) and cytochrome C (**B**) in HeLa cells. Cells were treated with various concentrations of γ-tocotrienol for 24 h. The expression levels of the proteins were analyzed through Western blot assay. * *p* < 0.05, ** *p* < 0.01, compared to the negative control group (*n* = 3). M: Marker; B: Blank control; C: Ethanol control.

To distinguish between the two main apoptotic pathways of HeLa cells induced by γ-tocotrienol, the expression of caspase-8 and caspase-9 were investigated by Western blotting. γ-tocotrienol-treated HeLa cells did not exhibit a significant increase in caspase-8 activity (Figure 8D). The expression of cleaved caspase-9 was induced in HeLa cells treated with 30, 45 and 60 μM γ-tocotrienol (Figure 9A).

During the initiation phase of apoptosis via the mitochondria-mediated death pathway, cytochrome c of mitochondria is released into the cytosol as a key event [34]. We also detected cytochrome c protein levels in both the mitochondrial and cytosolic fractions in HeLa cells treated with γ-tocotrienol for 24 h (Figure 9B). These results suggest that γ-tocotrienol specifically promoted cytochrome c release from the mitochondria into the cytosol of HeLa cells.

## 3. Discussion

Cervical cancer is the fourth most common cancer among women worldwide, with an estimated 528,000 new cases and 266,000 deaths annually [35]. It is the leading cause of cancer mortality among women in developing countries [36]. Cervical cancer is particularly amenable to prevention as it has a long pre-clinical phase and the natural history of cervical carcinogenesis is well researched. It has been reported that several phytochemicals, including phenolics, flavonoids and carotenoids from vegetables, grains, fruits, and other plant products can interfere with cell regulation and proliferation, being involved in multiple signaling pathways that are disrupted during tumor initiation, proliferation and propagation [37,38]. γ-tocotrienol is a phenolic that has attracted great attention for its potential as a chemopreventive and chemotherapeutic agent in various types of cancer cells. However, the molecular mechanisms responsible for the anti-proliferative action of tocotrienols are not entirely understood, especially in gynecology tumors.

In our study, γ-tocotrienol at 30–60 μM significantly inhibited HeLa human cervical cancer cell proliferation in a dose- and time- dependent manner, and no cytotoxicity was observed at low concentrations (15 μM). The IC_50_ of γ-tocotrienol for inhibition of HeLa human cervical cancer cell proliferation was 59.10, 46.90 and 18.40 μM at 12, 24 and 48 h. Previous studies showed that treatment with γ-tocotrienol at 100 μM and above for 24 h, effectively inhibited the growth of cervical cancer CaSki cells with IC_50_ value of 75 μM [39]. However, treatment with 3 μM γ-tocotrienol for 24 h exerted inhibitory effect on the proliferation of HeLa cell in a time-dependent and dose-dependent manner, IC_50_ values were 2.85 ± 0.07 μM [32]. These results were not completely inconsistent with our findings. Meantime, the 50% inhibitory concentrations for γ-tocotrienol in our study are higher or lower than those for other cell lines, such as 7.4 μM (3 days) in human hepatocellular carcinoma cells [40], less than 10 µM (4 days) using MCF-7 and MDA-MB-231 human mammary cells [41], 31.40 ± 1.51 µM (24 h) in SW620 cells and 32.69 ± 1.29 µM (24 h) in HCT-8 cells [42], 17.50 μM (2 days) in human colon cancer HCT116 cells [43], 35.45 μM (2 days) in human colon cancer HT-29 cells [24]. Another study showed that the IC_50_ values of γ-tocotrienol were 336 μM and 141 μM (1 day) in HT-29 and SW837 human colorectal cancer cells [25]. The differences may be due to the time of treatment, cell numbers seeded, state of the cells, operators, or varieties of cell line. In the present study, we also demonstrated that the mitotic index was decreased compared with the control group as the incubation time and concentration of γ-tocotrienol increased. Moreover, γ-tocotrienol significantly inhibited colony formation in HeLa cells in a dose-and time-dependent manner (*p* < 0.05) when the concentration of γ-tocotrienol was higher than 15 μM. PCNA, a nuclear protein that binds to DNA polymerase, is a cell-cycle regulator expressed in the nucleus of proliferating cells. Ki-67 protein expression occurs during the G_1_ phase, increases during the cell cycle, and rapidly declines after mitosis. PCNA and Ki-67 are often used as a proliferation marker [44,45]. In this study, γ-tocotrienol was demonstrated to down-regulate the protein expression of Ki-67 and PCNA of HeLa cells. Our data suggested a specific antiproliferative activity for γ-tocotrienol toward HeLa human cervical cancer cells.

To test the effect of γ-tocotrienol on the proliferation of HeLa cells further, cell-cycle distribution experiments were conducted. Our results showed that treatment with above 30 μM of γ-tocotrienol for 12 and 24 h induce the G0/G1 arrest and decreased numbers in S phase in HeLa cells compared with the control group. It has been reported that treatment with 1–3 μM of γ-tocotrienol for 24 h induces G0/G1 phase arrest and resulted decline in the percentage of cells in the S phase in a dose-dependent manner in HeLa cells [32]. Our previous data showed that γ -tocotrienol at 30–60 μM affected the cell-cycle distribution in HT-29 cells, resulting in appreciable arrest in the G0/G1 phase and S phase decreased at 48 h [24]. Previous studies also showed that treatment with γ-tocotrienol (10–30 μM) for 12 h induced G0/G1 phase arrest in human leukemia HL-60 cells [46]. Murine melanoma B16 cells treated with 20 μM of γ-tocotrienol for 3 h were arrested in the G1 phase and decreased the proportion of the cells in S phase [47]. The difference in dose of γ-tocotrienol may be associated with the time of treatment, cell numbers seeded, varieties of cell line, cell viability, inoculation density, or laboratory conditions. Our results suggested that γ-tocotrienol-induced G0/G1 phase arrest might be involved in the inhibition of HeLa cell proliferation.

Apoptosis is the mechanism used by metazoans to regulate tissue homeostasis through the elimination of redundant or potentially deleterious cells. Apoptosis induction is arguably the most potent defense against cancer. It is well known that certain chemopreventive agents can induce apoptosis in tumor cells [48]. It has also been reported that γ-tocotrienol can induce MDA-MB-231 [18], HT-29 [24] and SGC-7901 [31] cell apoptosis. In our present studies, morphology and flow cytometry demonstrated that treatment with 30–60 μM of γ-tocotrienol induced apoptosis in HeLa cells and these effects were dose- and time-dependent. Two important groups of proteins involved in apoptotic cell death are members of the Bcl-2 family [49] and a class of cysteine proteases known as caspases [50]. The Bcl-2 family proteins are key regulators of apoptosis, which include both anti-apoptotic (Bcl-2) and pro-apoptotic proteins (Bax), and a slight change in the dynamic balance of these proteins may result either in inhibition or promotion of cell death [51]. The Bcl-2 family proteins have been reported to regulate apoptosis by controlling the mitochondrial membrane permeability [52]. A decrease in the ratio of Bcl-2/Bax stimulates the release of cytochrome c from mitochondria into the cytosol. Caspase-3 can be activated through cytosolic release of cytochrome c [53]. Activated caspase-3 leads to PARP cleavage. Usually the cleavage of PARP is used as an indicator of apoptosis. In the study, we found that γ-tocotrienol up-regulated the expression of Bax, down-regulated the expression of Bcl-2, induced the cleavage of 32 kDa procaspase-3 into its active 20 kDa form, and resulted in subsequent PARP cleavage in HeLa cell. These results suggested that Bcl-2 and Bax participated in the regulation of apoptosis induction in HeLa cells by γ-tocotrienol. Similar results have been obtained in Hep3B [29], HT-29 [24] and SGC-7901 [30] cells. However, expression of Bax and Bcl-2 (mRNA and protein) did not change significantly, PARP cleavage was not detectable in MDA-MB-231 cells treated with 25 μg/mL (≈ 60.89 μM) γ-tocotrienol for 4, 8, 12, 18 and 24 h [18]. Apoptosis could be triggered through the activation of either an extrinsic (death receptors) pathway or an intrinsic (mitochondrial) pathway, which are initiated by caspase-8 and caspase-9, respectively [54,55]. Caspase-8 is crucial for triggering apoptosis via death receptors since its recruitment to and activation at the death-inducing signaling complex is the decisive step for the initiation of the caspase cascade leading to apoptosis [56]. In contrast, the mitochondrial pathway requires the release of mitochondrial cytochrome c and the formation of a large multiprotein complex comprising cytochrome c, Apaf-1 and procaspase-9. Caspase-8 and caspase-9 will then proteolytically activate downstream caspases, in particular caspases-3 and -7, which are responsible for the apoptotic destruction of the cell [31]. In the present study, caspase-9, the apical caspase in mitochondria-mediated apoptotic pathway, but not caspase-8, was activated during the process of apoptosis induced by γ-tocotrienol in HeLa cells. We also noticed that γ-tocotrienol induced the release of cytochrome c from the mitochondria into the cytosol in HeLa cells. Hence, we confirmed that γ-tocotrienol induces the expression of Bcl-2 family proteins and increases the release of cytochromec, which then leads to activation of procaspase-9 and caspase-3 to induce fragmentation of PARP. In other words, γ-tocotrienol-induced apoptosis in HeLa cells involved the mitochondria-mediated apoptotic pathway. 

In conclusion, the present study indicated that γ-tocotrienol dose- and time- dependently inhibited cell proliferation and induced apoptosis of HeLa cells through arresting cell cycle at the G0/G1 phase, increasing the Bax/Bcl-2 ratio, the activation of caspase-3 and caspase-9, and cleavage of PARP. γ-tocotrienol promotes apoptosis via a mitochondria-mediated intrinsic apoptotic pathway, independent of death receptor signaling. Additionally, the findings provide good evidence for the anti-cancer effect of γ-tocotrienol, and suggest that γ-tocotrienol is a good potential natural compound for the chemoprevention and treatment of cervical cancer.

## 4. Materials and Methods

### 4.1. Materials

Human cervical cancer HeLa cell line was obtained from the Department of Pathophysiology, Basic Medical, Jiamusi University (Jiamusi, China). γ-tocotrienol was purchased from Cayman Chemicals CO., Ltd. (Ann Arbor, MI, USA). The 3-(4,5-dimethylthiazol-2-yl)-2,5-diphenyltetrazolium bromide (MTT) and DAPI Staining kit were purchased from Sigma Aldrich (Kansas, MO, USA). Rabbit polyclonal antibodies for glyceraldehyde-3-phosphate dehydrogenase (GAPDH) (sc-25778), caspase-3 (sc-7148), caspase-9 (sc-8355), and PRAP-1 (sc-7150) were bought from Santa Cruz Biotechnology (Santa Cruz, CA, USA). Mouse monoclonal antibody for Bax (sc-7480), Bcl-2 (sc-7382), caspase-8 (sc-5263), and anti-cytochrome c (sc-13156) were obtained from Santa Cruz Biotechnology (Santa Cruz, CA, USA). Goat anti-rabbit (w3960) and anti-mouse (w3950) secondary antibodies were purchased from Promega (Madison, WI, USA).

### 4.2. Cell Culture

Human cervical cancer HeLa cells were cultured in RPMI-1640 (Gibco, Paisley, Scotland) containing 10% (*v*/*v*) heat-inactivated fetal bovine serum (Gibco), maintained at 37 °C in a humidified incubator with 5% CO_2_ and 95% air. Cells were plated in 75-cm^2^ flasks and allowed to grow to approximately 70% confluence before experimentation.

### 4.3. Cell Viability

The effect of γ-tocotrienol on cell viability was determined by an MTT assay. Briefly, exponentially growing cells were seeded in 24-well plates (Nunc, Wiesbaden, Germany) at 2.0 × 10^4^ cells/well. After 24 h of incubation, the medium was removed and the cells were treated with 1000 μL of medium containing various concentrations (15, 30, 45, and 60 μM) of γ-tocotrienol for the desired time. Negative control cells were supplemented with 0.15% ethanol vehicle. Each concentration of γ-tocotrienol was repeated in three wells. 100 μL of MTT (5 mg/mL in PBS) (Gibco, Rockville, MD, USA) was added to each well and incubated at 37 °C for 4 h. The medium was carefully removed and 750 μL of dimethyl sulfoxide (DMSO) was added to each well. The plates were shaken for 10 min and the absorbance at 490 nm was measured in an 550 Universal microplate reader (Bio-Tek Instruments, Inc., Winooski, VT, USA). Growth inhibition by γ-tocotrienol was calculated as percentage of cell viability, taking the viability of the blank control cells as 100%.

### 4.4. Mitotic Index

Exponentially growing HeLa cells were seeded in 24-well plates at 1.0 × 10^4^ cells/well and incubated for 24 h to allow the cells to attach the bottom of plate, and then the medium was removed and the cells were treated with 15, 30, 45, and 60 μM of γ-tocotrienol for 12, 24 and 48 h. Each concentration of γ-tocotrienol was repeated in three wells. After incubation, the cells were fixed with methanol and stained with 1% Giemsa. The number of cells in mitosis was counted among 1000 cells under an inverted microscope (Olympus CK40, Tokyo, Japan). The mitotic index was calculated using the following Equation (1):

mitotic index = number of mitotic cells/total number of cells × 100%(1)

### 4.5. Colony Formation

Exponentially growing cells were plated at 200 cells/well in 24-well plates and allowed to attach for 24 h. The medium was removed and the cells were treated with 15, 30, 45 and 60 μM of γ-tocotrienol. Each concentration of γ-tocotrienol was repeated in three wells. After 12, 24 or 48 h of incubation, the cells were fixed with methanol and stained with Giemsa. Colonies containing more than 50 cells originating from single cells were counted under the inverted microscope (Olympus CK40).

### 4.6. Flow Cytometric Analysis

Exponentially growing HeLa cells were plated at a density of 5.5 × 10^5^ cells per 100-mm culture dish for 24 h. The medium was removed and the cells were treated with 15, 30, 45 and 60 μM of γ-tocotrienol for 12 and 24 h, then harvested and washed twice with PBS. The cells were then resuspended in 150 μL PBS and fixed in 85% ice-cold ethanol for 2 h at 4 °C. They were then centrifuged at 1000 rpm for 5 min and resuspended with PBS, repeated twice. Finally, each samples added 0.5 mL PI (5 μg/mL), maintained at 37 °C in a humidified incubator for 30 min. Data acquisition was done on a Guava easyCyte Flow Cytometer (Guava Technologies, Billerica, MA, USA) and analyzed by Modfit L T software (Verity Software House Inc., Topsham, ME, USA).

### 4.7. Morphologic Observation of Apoptosis

After treatment with γ-tocotrienol, morphologic changes in HeLa cells were assessed by inverted microscope, fluorescence microscopy and transmission electron microscopy. Briefly, the cells were treated with 15, 30, 45 and 60 μM of γ-tocotrienol for 12, 24 and 48 h and observed under inverted microscopy. Changes in the nuclei were investigated by staining the cells with fluorescent DNA-binding dyes (DAPI). Cells were harvested and washed twice with PBS, and 25 μL of cell suspension was mixed with 1 μL of dyeing solution (4′,6-diamidino-2-phenylindole, DAPI). Nuclear morphology was assessed by fluorescence microscopy (Olympus IX70, Tokyo, Japan). For transmission electron microscopy, the cells were harvested and fixed with 600 μL glutaraldehyde, the pellets were dehydrated in graded ethanol solutions and embedded in Epon 812. Ultrathin sections of pellet were counterstained with uranyl acetate and lead citrate and observed under a JEM-1220 transmission electron microscope (JEOL Ltd., Tokyo, Japan).

### 4.8. Detection of DNA Fragmentation

The agarose gel electrophoresis method described previously was modified to determine DNA fragmentation [57,58]. Briefly, HeLa cells were treated with 15, 30, 45 and 60 μM of γ-tocotrienol for 12, 24 and 48 h, and then washed twice with PBS. Total DNA was isolated using a Apoptotic DNA Ladder Kit (Applygen Technologies Inc., Beijing, China). DNA agarose electrophoresis was executed at 60 V on a 1.2% agarose gel. Loading was conducted with 5 μL samples with 1 μL 6× sample buffer, and 5 μL DNA marker. The gel was photographed under ultraviolet illumination.

### 4.9. Western Blot Analysis

The HeLa cells were treated with various concentrations of γ-tocotrienol for 12 h and then collected, washed twice with PBS, and detached in PBS containing 0.25% pancreatin. Whole-cell lysates obtained from the different treatment groups were isolated by lysing in 20 mM Tris-HCl, pH 7.5, 2% SDS (*w*/*v*), 2 mM benzamidine, 0.2 mM phenyl-methane-sulphonyl fluoride. Preparation of mitochondrial and cytosolic fractions was conducted as previously described [18]. The total protein concentrations of each sample were measured in a 550 Universal microplate reader (Bio-Tek Instruments, Inc.) at 562 nm. For western blotting, 100 μg of protein was resolved on 10% polyacrylamide gels and transferred to a nitrocellulose membrane. The membrane was blocked in blocking buffer (1% BSA, 1% Tween 20 in 20 mM Tris-buffered saline (TBS), pH 7.6) for 30 min at 37 °C in a hybridization oven, incubated with appropriate monoclonal or polyclonal primary antibody in blocking buffer for 2 h at 37 °C or overnight at 4 °C. The membrane was washed 3 × 5 times with Tris-buffered saline tween-20 (TBST) followed by incubation with anti-mouse or anti-rabbit secondary antibody at 37 °C for 1 h. The membrane was washed 3 × 5 times with TBST and then washed with TBS twice. Then the membrane was incubated with alkaline phosphatase until an appropriate signal level was obtained. Protein bands were detected by FluorChem Imaging Systems (Bio-Rad, Hercules, CA, USA).

### 4.10. Statistical Analysis

Statistical analysis was performed using SPSS version 14.0 (SPSS, Inc., Chicago, IL, USA). The data were expressed as Mean ± SD. Differences between the control and treated groups were evaluated by the one-way analysis of variance (ANOVA) test with the Bonferroni post hoc multiple comparisons and considered significant at *p <* 0.05.

## Figures and Tables

**Table 1:** Effect of γ-tocotrienol on the mitotic index of HeLa cells (*n* = 3).

Groups (μM)	No. of Cancer Cells in Mitosis (Mean ± SD)			Mitotic Index (%)			Inhibition (%)		
	12 h	24 h	48 h	12 h	24 h	48 h	12 h	24 h	48 h
0	214.00 ± 4.20	256.00 ± 3.70	293.00 ± 3.50	21.40	25.60	29.30	−	−	−
ethanol	216.00 ± 6.50	252.00 ± 4.50	287.00 ± 3.80	21.60	25.20	28.70	−0.90	1.60	2.00
15	219.00 ± 2.50	268.00 ± 3.60	313.00 ± 3.20	21.90	26.80	31.30	−2.30	−4.70	−6.80
30	195.60 ± 6.20	222.40 ± 2.90	236.00 ± 5.30	19.60	22.20	23.60 *	8.40	13.10	19.50
45	159.00 ± 5.30	182.00 ± 4.20	80.00 ± 6.70	15.90 *	18.20 *	0.80 **	25.70	28.90	72.70
60	137.00 ± 5.00	102.00 ± 6.30	61.00 ± 5.90	13.70 *	10.20 *	0.60 **	36.00	60.20	79.20

* *p* < 0.05, ** *p* < 0.01 compared to the control group.

**Table 2:** Effect of γ-tocotrienol on colony formation in HeLa cells (*n* = 3).

Groups (μM)	No. of Cancer Cells Colonies (Mean ± SD)			Colony Formation (%)			Colony Inhibition (%)		
	12 h	24 h	48 h	12 h	24 h	48 h	12 h	24 h	48 h
0	76.30 ± 2.90	79.80 ± 4.30	84.30 ± 3.80	38.15	39.90	42.15	−	−	−
ethanol	76.00± 1.00	77.00 ± 3.00	83.50 ± 3.00	38.00	38.50	41.75	0.40	3.50	−0.90
15	79.00 ± 4.00	84.30 ± 3.30	90.30 ± 1.80	39.50	42.30	45.15	−3.50	−5.60	−7.10
30	70.50 ± 6.80	56.00 ± 3.00	41.80 ± 2.30	35.25	28.00 *	20.90 *	7.60	29.80	50.40
45	34.00 ± 3.00	8.50 ± 2.00	0.50 ± 0.50	17.00 **	4.30 **	0.25 **	55.40	89.30	99.40
60	0.30 ± 0.40	0	0	0.20 **	0 **	0 **	99.60	100	100

* *p* < 0.05, ** *p* < 0.01, compared to the control group.

**Table 3:** Impact of γ-tocotrienol on the distribution of HeLa cell cycle on 12 h (*n* = 3).

Group (μM)	Cell Cycle Distribution (%)			Cell Count
	G0/G1	S	G2/M	× 10^6^
0	56.27 ± 3.50	26.14 ± 2.81	17.59 ± 1.10	1.67
ethanol	56.95 ± 2.67	23.77 ± 1,83	19.28 ± 3.47	1.70
15	55.17 ± 4.22	24.22 ± 2.58	20.61 ± 2.32	1.79
30	61.27 ± 2.73	19.84 ± 3.21	18.89 ± 2.18	1.33
45	68.13 ± 3.20 *	14.90 ± 2.10 *	16.97 ± 2.51	1.04
60	72.03 ± 3.48 *	8.88 ± 1.71 **	19.09 ± 3.21	0.82

* *p* < 0.05, ** *p* < 0.01, compared to the control group.

**Table 4:** Impact of γ-tocotrienol on the distribution of HeLa cells cycle on 24 h (*n* = 3).

Group (μM)	Cell Cycle Distribution (%)			Cell Count
	G0/G1	S	G2/M	× 10^6^
0	57.57 ± 3.50	32.43 ± 2.81	10.00 ± 1.10	2.13
ethanol	55.97 ± 2.67	34.03 ± 1.83	12.81 ± 3.47	2.15
15	53.16 ± 4.22	36.02 ± 2.58	10.08 ± 2.32	2.27
30	63.75 ± 2.73 *	27.14 ± 3.21 *	9.11 ± 2.18	1.49
45	70.31 ± 3.20 *	19.83 ± 2.10 **	9.86 ± 2.51	1.19
60	75.87 ± 3.48 *	15.92 ± 1.71 **	8.21 ± 3.21	0.62

* *p* < 0.05, ** *p* < 0.01, compared to the control group.

## References

[1] Siegel R., Ward E., Brawley O., Jemal A. (2011). Cancer statistics, 2011: The impact of eliminating socioeconomic and racial disparities on premature cancer deaths. CA Cancer J. Clin..

[2] Global Cancer Rates Could Increase by 50% to 15 Million by 2020. http://www.who.int/mediacentre/news/releases/2003/pr27/en/.

[3] Doll R., Peto R. (1981). The causes of cancer: Quantitative estimates of avoidable risks of cancer in the United States today. J. Natl. Cancer Inst..

[4] Willett W.C. (2002). Balancing life-style and genomics research for disease prevention. Science.

[5] DiMarco-Crook C., Xiao H. (2015). Diet-based strategies for cancer chemoprevention: The role of combination regimens using dietary bioactive components. Annu. Rev. Food Sci. Technol..

[6] Key T.J., Schatzkin A., Willett W.C., Allen N.E., Spencer E.A., Travis R.C. (2004). Diet, nutrition and the prevention of cancer. Public Health Nutr..

[7] Thomasset S.C., Berry D.P., Garcea G., Marczylo T., Steward W.P., Gescher A.J. (2007). Dietary polyphenolic phytochemicals-promising cancer chemopreventive agents in humans? A review of their clinical properties. Int. J. Cancer.

[8] Ling M.T., Luk S.U., Al-Ejeh F., Khanna K.K. (2011). Tocotrienol as a potential anticancer agent. Carcinogenesis.

[9] Sen C.K., Khanna S., Rink C., Roy S. (2007). Tocotrienols: The emerging face of natural vitamin E. Vitam. Horm..

[10] Sen C.K., Khanna S., Roy S. (2006). Tocotrienols: Vitamin E beyond tocopherols. Life Sci..

[11] Nesaretnam K., Guthrie N., Chambers A.F., Carroll K.K. (1995). Effect of tocotrienols on the growth of a human breast cancer cell line in culture. Lipids.

[12] Qureshi A.A., Sami S.A., Salser W.A., Khan F.A. (2002). Dose-dependent suppression of serum cholesterol by tocotrienol-rich fraction (TRF25) of rice bran in percholesterolemic humans. Atherosclerosis.

[13] Khanna S., Roy S., Ryu H., Bahadduri P., Swaan P.W., Ratan R.R., Sen C.K. (2003). Molecular basis of vitamin E action: tocotrienol modulates 12-lipoxygenase, a keymediatorof glutamate-induced neurodegeneration. J. Biol. Chem..

[14] He L., Mo H., Hadisusilo S., Qureshi A.A., Elson C.E. (1997). Isoprenoids suppress the growth of murine B16 melanomas in vitro and in vivo. J. Nutr..

[15] Liu H.K., Wang Q., Li Y., Sun W.G., Liu J.R., Yang Y.M., Xu W.L., Sun X.R., Chen B.Q. (2010). Inhibitory effects of γ-tocotrienol on invasion and metastasis of human gastric adenocarcinoma SGC-7901 cells. J. Nutr. Biochem..

[16] Yu W., Simmons-Menchaca M., Gapor A., Sanders B.G., Kline K. (1999). Induction of apoptosis in human breast cancer cells by tocopherols and tocotrienols. Nutr. Cancer.

[17] Sylvester P.W., McIntyre B.S., Gapor A., Briski K.P. (2001). Vitamin E inhibition of normal mammary epithelial cell growth is associated with a reduction in protein kinase C(alpha) activation. Cell Prolif..

[18] Takahashi K., Loo G. (2004). Disruption of mitochondria during tocotrienol-induced apoptosis in MDA-MB-231 human breast cancer cells. Biochem. Pharmacol..

[19] Mc Intyre B.S., Briski K.P., Gapor A., Sylvester P.W. (2000). Antiproliferative and apoptoticeffects of tocopherols and tocotrienols on preneoplastic and neoplastic mousemammary epithelial cells. Proc. Soc. Exp. Biol. Med..

[20] Mc Intyre B.S., Briski K.P., Tirmenstein M.A., Fariss M.W., Gapor A., Sylvester P.W. (2000). Antiproliferative and apoptotic effects of tocopherols and tocotrienols onnormal mouse mammary epithelial cells. Lipids.

[21] Wali V.B., Bachawal S.V., Sylvester P.W. (2009). Endoplasmic reticulum stress mediates gamma-tocotrienol-induced apoptosis in mammary tumor cells. Apoptosis.

[22] Shah S.J., Sylvester P.W. (2005). γ-tocotrienol inhibits neoplastic mammary epithelial cell proliferation by decreasing Akt and nuclear factor kappaB activity. Exp. Biol. Med..

[23] Abdul Rahman A., Jamal A.R., Harun R., Mohd Mokhtar N., Wan Ngah W.Z. (2014). Gamma-tocotrienol and hydroxy-chavicol synergistically inhibits growth and induces apoptosis of human glioma cells. BMC Complement. Altern. Med..

[24] Xu W.L., Liu J.R., Liu H.K., Qi G.Y., Sun X.R., Sun W.G., Chen B.Q. (2009). Inhibition of proliferation and induction of apoptosis by gamma-tocotrienol in human colon carcinoma HT-29 cells. Nutrition.

[25] Yusof K.M., Makpol S., Jamal R., Harun R., Mokhtar N., Wan Ngah W.Z. (2015). γ-Tocotrienol and 6-Gingerol in Combination Synergistically Induce Cytotoxicity and Apoptosis in HT-29 and SW837 Human Colorectal Cancer Cells. Molecules.

[26] Jiang Q., Rao X., Kim C.Y., Freiser H., Zhang Q., Jiang Z., Li G. (2012). Gamma-tocotrienol induces apoptosis and autophagy in prostate cancer cells by increasing intracellular dihydrosphingosine and dihydroceramide. Int. J. Cancer.

[27] Campbell S.E., Whaley S.G., Phillips R., Aggarwal B.B., Stimmel J.B., Leesnitzer L., Blanchard S.G., Stone W.L., Muenyi Christian, Krishnan K. (2008). Gamma tocotrienol and prostate cancer: the regulation of two independent pathways to potentiate cell growth inhibition and apoptosis. J. Oil Palm Res..

[28] Sakai M., Okabe M., Tachibana H., Yamada K. (2006). Apoptosis induction by γ-tocotrienol in human hepatoma Hep3B cells. J. Nutr. Biochem..

[29] Kani K., Momota Y., Harada M., Yamamura Y., Aota K., Yamanoi T., Takano H., Motegi K., Azuma M. (2013). γ-tocotrienol Enhances the Chemosensitivity of Human oral Cancer Cells to Docetaxel Through the Downregulation of the Expression of NF-kappa B-regulated Anti-apoptotic Gene Products. Int. J. Oncol..

[30] Sun W., Wang Q., Chen B., Liu J., Liu H., Xu W. (2008). γ-tocotrienol- induced apoptosis in human gastric cancer SGC-7901 cells is associated with a suppression in mitogen-activated protein kinase signalling. Br. J. Nutr..

[31] Sun W., Xu W., Liu H., Liu J., Wang Q., Zhou J., Dong F., Chen B. (2009). gamma-Tocotrienol induces mitochondria-mediated apoptosis in human gastric adenocarcinoma SGC-7901 cells. J. Nutr. Biochem..

[32] Wu S.J., Ng L.T. (2010). Tocotrienols inhibited growth and induced apoptosis in human HeLa cells through the cell cycle signaling pathway. Integr. Cancer Ther..

[33] Comitato R., Guantario B., Leoni G., Nesaretnam K., Ronci M.B., Canali R., Virgili F. (2016). Tocotrienols induce endoplasmic reticulum stress and apoptosis in cervical cancer cells. Genes Nutr..

[34] Budihardjo I., Oliver H., Lutter M., Luo X., Wang X. (1999). Biochemical pathways of caspase activation during apoptosis. Annu. Rev. Cell Dev. Biol..

[35] Cheikh A., El Majjaoui S., Ismaili N., Cheikh Z., Bouajaj J., Nejjari C., El Hassani A., Cherrah Y., Benjaafar N. (2016). Evaluation of the cost of cervical cancer at the National Institute of Oncology, Rabat. Pan. Afr. Med. J..

[36] Aggarwal P. (2014). Cervical cancer: Can it be prevented?. World J. Clin. Oncol..

[37] Priyadarsini R.V., Nagini S. (2012). Cancer chemoprevention by dietary phytochemicals: Promises and pitfalls. Curr. Pharm. Biotechnol..

[38] Lee K.W., Bode A.M., Dong Z. (2011). Molecular targets of phytochemicals for cancer prevention. Nat. Rev. Cancer.

[39] Hasani N.A., Yusoff P.A., Bak K., Mt A.G., Wan Ngah W.Z. (2008). The Possible Mechanism of Action of Palm oil Gamma-tocotrienol and Alpha-tocopherol on the Cervical Carcinoma CaSki Cell Apoptosis. Biomed. Res..

[40] Wada S., Satomi Y., Murakoshi M., Noguchi N., Yoshikawa T., Nishino H. (2005). Tumor suppressive effects of tocotrienol in vivo and in vitro. Cancer Lett..

[41] Tiwari R.V., Parajuli P., Sylvester P.W. (2014). γ-tocotrienol-induced autophagy in malignant mammary cancer cells. Exp. Biol. Med..

[42] Zhang J.S., Li D.M., Ma Y., He N., Gu Q., Wang F.S., Jiang S.Q., Chen B.Q., Liu J.R. (2013). γ-tocotrienol induces paraptosis-like cell death in human colon carcinoma SW620 cells. PLoS ONE.

[43] Yang Z.H., Xiao H., Jin H.Y., Koo P.T., Tsang D.J., Yang C.S. (2010). Synergistic Actions of Atorvastatin with Gamma-tocotrienol and Celecoxib Against Human Colon Cancer HT29 and HCT116 Cells. Int. J. Cancer.

[44] McCormick D., Chong H., Hobbs C., Datta C., Hall P.A. (1993). Detection of the Ki-67 antigen in fixed and wax-embedded sections with the monoclonal antibody MIB1. Histopathology.

[45] Barnouti Z.P., Owtad P., Shen G., Petocz P., Darendeliler M.A. (2011). The biological mechanisms of PCNA and BMP in TMJ adaptive remodeling. Angle Orthod..

[46] Chen C.C., Liu T.Y., Huang S.P., Ho C.T., Huang T.C. (2015). Differentiation and Apoptosis Induction by Lovastatin and γ-tocotrienol in HL-60 cells via Ras/ERK/NF-κB and Ras/Akt/NF-κB Signaling Dependent Down-regulation of Glyoxalase 1 and HMG-CoA reductase. Cell. Signal..

[47] Mo H., Elson C.E. (1999). Apoptosis and Cell-cycle Arrest in Human and Murine Tumor Cells are Initiated by Isoprenoids. J. Nutr..

[48] Sun S.Y., Hail N., Lotan R. (2004). Apoptosis as a novel target for cancer chemoprevention chemoprevention. J. Natl. Cancer Inst..

[49] Korsmeyer S.J. (1999). BCL-2 gene family and the regulation of programmed cell death. Cancer Res..

[50] Cohen G.M. (1997). Caspases: The executioners of apoptosis. Biochem. J..

[51] Ola M.S., Nawaz M., Ahsan H. (2011). Role of Bcl-2 family proteins and caspases in the regulation of apoptosis. Mol. Cell. Biochem..

[52] Tsujimoto Y., Shimizu S. (2000). Bcl-2 family: Life-or-death switch. FEBS Lett..

[53] Communal C., Sumandea M., de Tombe P., Narula J., Solaro R.J., Hajjar R.J. (2002). Functional consequences of caspase activation in cardiac myocytes. Proc. Natl. Acad. Sci. USA..

[54] Wajant H. (2002). The Fas signaling pathway: More than a paradigm. Science.

[55] Cho S.G., Choi E.J. (2002). Apoptotic signaling pathways: Caspases and stress-activated protein kinases. BMB Rep..

[56] Kantari C., Walczak H. (2011). Caspase-8 and bid: Caught in the act between death receptors and mitochondria. BBA-Mol. Cell Res..

[57] Sylte M.J., Corbeil L.B., Inzana T.J., Czuprynski C.J. (2001). Haemophilus somnus induces apoptosis in bovine endothelial cells in vitro. Infect. Immun..

[58] Lee Y.J., Yin H.Q., Kim Y.H., Li G.Y., Lee B.H. (2004). Apoptosis inducing effects of 6-methoxydihydrosanguinarine in HT29 colon carcinoma cells. Arch. Pharm. Res..

